# Relation between Approximate Number System Acuity and Mathematical Achievement: The Influence of Fluency

**DOI:** 10.3389/fpsyg.2016.01966

**Published:** 2016-12-20

**Authors:** Li Wang, Yuhua Sun, Xinlin Zhou

**Affiliations:** ^1^State Key Laboratory of Cognitive Neuroscience and Learning, Beijing Normal UniversityBeijing, China; ^2^Siegler Center for Innovative Learning, Advanced Innovation Center for Future Education, Beijing Normal UniversityBeijing, China; ^3^Institute of Education Science, Xinjiang Normal UniversityUrumqi, China

**Keywords:** visual form perception, approximate number system, mathematical achievement, mathematical fluency, subtraction

## Abstract

Previous studies have observed inconsistent relations between the acuity of the Approximate Number System (ANS) and mathematical achievement. In this paper, we hypothesize that the relation between ANS acuity and mathematical achievement is influenced by fluency; that is, the mathematical achievement test covering a greater expanse of mathematical fluency may better reflect the relation between ANS acuity and mathematics skills. We explored three types of mathematical achievement tests utilized in this study: Subtraction, graded, and semester-final examination. The subtraction test was designed to measure the mathematical fluency. The graded test was more fluency-based than the semester-final examination, but both involved the same mathematical knowledge from the class curriculum. A total of 219 fifth graders from primary schools were asked to perform all three tests, then given a numerosity comparison task, a visual form perception task (figure matching), and a series of other tasks to assess general cognitive processes (mental rotation, non-verbal matrix reasoning, and choice reaction time). The findings were consistent with our expectations. The relation between ANS acuity and mathematical achievement was particularly clearly reflected in the participants’ performance on the visual form perception task, which supports the domain-general explanations for the underlying mechanisms of the relation between ANS acuity and math achievement.

## Introduction

The Approximate Number System (ANS) is responsible for estimating numbers of objects (Feigenson et al., 2004). The ability to navigate the ANS, or “ANS acuity”, has been demonstrated in animals (Dehaene et al., 1998; Brannon, 2006) and emerges early in human development (Xu and Spelke, 2000; Xu, 2003). ANS acuity is considered to evolve due to its adaptive value; naturally, it assists in hunting, gathering, territorial marking, and other survival activities (Pica et al., 2004; Halberda et al., 2008). For modern humans, ANS acuity is crucial for success in education and employment.

Several studies have shown that ANS acuity is related to mathematical achievement. ANS acuity in childhood, for example, is correlated with mathematical performance (Halberda et al., 2008; Mundy and Gilmore, 2009; Inglis et al., 2011; Libertus et al., 2011; Halberda et al., 2012; Bonny and Lourenco, 2013; Fuhs and McNeil, 2013; Chen and Li, 2014; Fazio et al., 2014). For example, Fuhs and McNeil (2013) found that ANS acuity accounts for significant variance in mathematical achievement after controlling for receptive vocabulary in preschoolers ranging in age from 44 to 71 months. ANS acuity measured in preschool or kindergarten can predict later mathematics performance, as well (De Smedt et al., 2009; Mazzocco et al., 2011; Libertus et al., 2013). Libertus et al. (2013) found that a child’s early ANS acuity predicts his or her math ability 6 months later, even when controlling for individual differences in age, expressive vocabulary, and math ability at the initial testing. In addition, positive relation between ANS acuity and mathematical achievement has been reported in adults. College students’ ANS acuity is related to their quantitative SAT (Scholastic Aptitude Test) scores (Lyons and Beilock, 2011; DeWind and Brannon, 2012; Libertus et al., 2012).

Other researchers failed to identify the relation between ANS acuity and mathematical performance throughout childhood and adulthood, however, (Inglis et al., 2011; Castronovo and Gobel, 2012; Price et al., 2012; Sasanguie et al., 2012; Vanbinst et al., 2012; Zhou and Cheng, 2015; Zhou et al., 2015). For example, Vanbinst et al. (2012) found that children’s ANS acuity is not associated with their performance on a curriculum-based, standardized mathematical achievement test, including multi-digit calculation, word problem solving, and geometry. Inglis et al. (2011) used the Woodcock Johnson III Tests of Achievement to test the mathematical achievement of adults and found no significant relationship between ANS acuity and any measure of math skills.

Previous studies have shown that the ANS acuity is typically associated with subtraction but not with mathematical problem-solving (e.g., Nunes et al., 2012; Wei et al., 2012b; Zhang et al., 2016). For example, Zhang et al. (2016) found that ANS acuity can predict the variance in subtraction but not the variance in mathematical reasoning measured with a number series completion task. The task of subtraction was usually used in the previous studies to measure the mathematical fluency (e.g., Geary et al., 2000; Inglis et al., 2011; Lyons and Beilock, 2011; Wei et al., 2012b; Zhou and Cheng, 2015; Zhou et al., 2015). The inconsistent findings regarding the relation between ANS acuity and mathematical achievement in previous studies are likely due to the differing extent of mathematical fluency measured via mathematical achievement tests. Math fluency typically reflects how fast and accurate students retrieve math facts and perform routine procedures; the student is dependent on step-by-step strategies in solving individual math problems, however. A mathematical achievement test covering mathematical fluency might show a closer relation between ANS acuity and mathematical achievement where problem-solving tests would show no such relation.

The aim of the current study was to investigate whether the relation between ANS acuity and mathematical achievement is influenced by fluency. Three types of mathematical achievement tests were applied: Subtraction, graded mathematical achievement test (from first to 12th grades), and a semester-final examination. Subtraction test are typically applied to measure mathematical fluency (Geary et al., 2000; Inglis et al., 2011; Wei et al., 2012b; Zhou and Cheng, 2015; Zhou et al., 2015), thus, it was expected that there would be association between ANS acuity and subtraction performance. The semester-final examination is typically designed by the local educational administrative department and administered to assess the students’ level of acquired mathematical knowledge over the course of the previous semester. The graded mathematical achievement test and semester-final examination involve the same mathematical knowledge from the course curriculum; the difference between the two is that the questions for the graded test involve semester-final examinations from the first to 12th grade level. Participants performed the test beginning with questions from the lowest (first) grade, and move on to higher grade questions as they provide correct answers; if they fail to answer higher grade questions, they are presented with lower grade questions again. For the semester-final examination, students used their newly acquired math knowledge to answer questions relevant to their most recent coursework; for the graded test, students used their comprehensive (and more fluency-based) math knowledge accumulated over several years of study. Thus, ANS acuity is expected to have little association with the mathematical achievement measured by the semester-final examination, but substantial association with that measured by the graded test.

The underlying mechanisms for the relation between ANS acuity and mathematical performance could be domain-specific or domain-general. Domain-specific explanations indicate that number sense or quantity processing in the ANS is attributed to the close relationship between the ANS and mathematical performance (Halberda et al., 2008). Domain-general explanations assert that the relation between ANS acuity with mathematical achievement is influenced by general cognitive factors. Recently, a visual form perception hypothesis has been proposed to explain the relation between ANS acuity and mathematical achievement (Zhou and Cheng, 2015; Zhou et al., 2015). A geometric figure-matching task was used to fully measure visual form perception, which involves the abilities to attend to and to identify distinguishing features and details of a figure (Zhou and Cheng, 2015; Zhou et al., 2015). In the task, each figure is a combination of two simple geometric figures (e.g., triangle, square, circle) that can be treated as a form or shape consisting of abstract lines. The participants judges whether a figure on the left side of the screen is the same as any of the figures on the right side, which are presented for 400 milliseconds (Zhou and Cheng, 2015; Zhou et al., 2015). Zhou et al. (2015) found no close relation between ANS and mathematical fluency when controlling for the figure matching score as well as the scores on other general cognitive measures (Raven’s Progressive Matrices, mental rotation, choice reaction, visual tracing, and digit span) in third- to fifth-graders (Zhou et al., 2015). Zhou and Cheng (2015) explored differences in geometric figure discrimination ability between children with dyscalculia and typically developed children; said differences were found to be representative of varying ANS acuity among the children. These results suggest that figure discrimination ability may account for the relation between ANS acuity and mathematical fluency; that is, the positive relation between ANS acuity and mathematical fluency is characterized by visual form perception. Participants who quickly and adeptly answer figure-matching questions can directly retrieve the forms involved in math fluency tasks from their stored memory (Zhou and Cheng, 2015; Zhou et al., 2015). In this study, we aimed to support the domain-specific explanation by testing whether visual form perception accounts for the relation between ANS acuity and mathematical achievement.

## Materials and Methods

### Participants

A total of 219 participants (128 male, 91 female, mean age = 11.5, *SD* = 0.6) were recruited from the fifth grade of primary schools in the Xinjiang autonomous region, P.R. China. The children were of various ethnicities including Han (108), Uyghur (77), Hui (28), and Kazak (6). All participants were taught math in Mandarin. They had normal or corrected-to-normal vision. Their parents provided written consent for their participation.

### Tests

**FIGURE 1:**
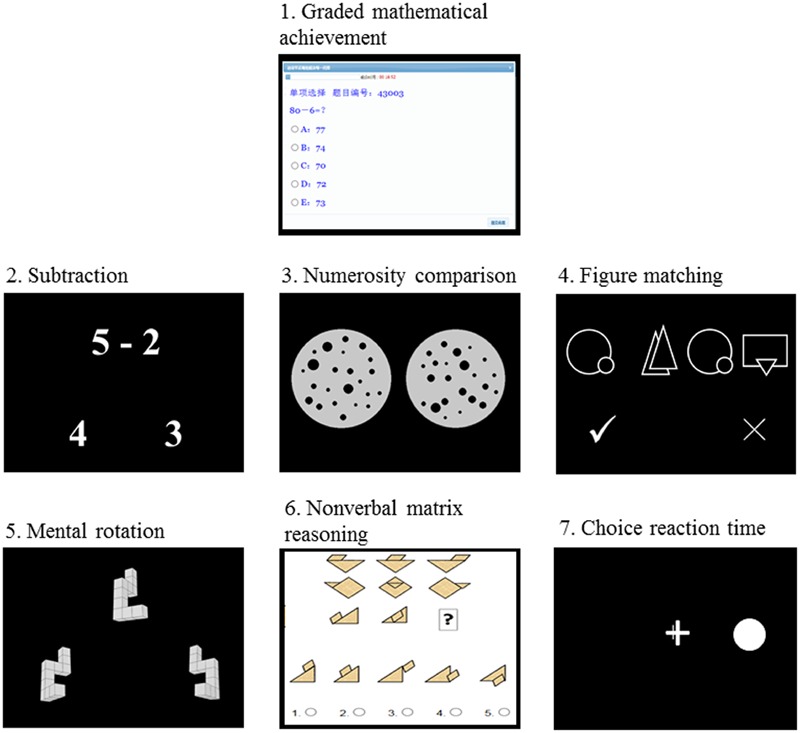
**Schematic representation of tests used in the current study (except semester-final examination test)**.

A total of eight tests were administered. All except the semester-final examination test were computerized with the Online Psychological Experiment System (OPES)^1^ (Wei et al., 2012b; Zhou et al., 2015). The illustration of trials for the different tests is shown in **Figure 1**. All except the choice reaction time task were time-limited.

#### Graded Mathematical Achievement

This test was designed as a standardized achievement test on which the child was asked to solve as many items as possible within 18 min. The questions used in the test were edited according to semester-final examinations, including number knowledge, operations, simple arithmetic, word problems, measurement, and geometry.

The questions for each grade were randomly chosen and grouped into five sets, each including three questions.

Participants were first given a set of questions from the first grade level. If the child correctly solved at least two questions from the set of three, the difficulty level was increased by one grade. If they gave incorrect answers for two questions in one set, the difficulty level stayed at the same grade. If they gave incorrect answers for all three questions in one set or failed to solve all five sets of questions, the difficulty level dropped to a lower grade (provided a lower grade was available). The test would stopped when the time was up or if all five sets of questions in the first grade were answered. Thus, different participants can receive questions according to theirs math skill, which were mainly below theirs grade level (93.4%). The final score was calculated as the sum of weighted scores in each grade, which was the number of correctly answered questions times the grade level (1–12). There were a total of 1,722 problems in the test database.

#### Semester-Final Examination

The current study used participants’ mathematics scores from their final term examinations as-provided by the participating primary schools.

The achievement test was developed by the Instruction Research Unit affiliated with the local Department of Education. It was administered to all students in the district at the end of each semester and covered the math knowledge acquired throughout the whole semester. The test involved number knowledge, operations, simple arithmetic, word problems, measurement, and geometry. Students had 90 min to complete this test.

#### Subtraction

There were 92 trials in total for this task, which included simple subtraction problems such as “6–2” and “16–8”; the minuends were 18 or smaller and the answers were all single-digit numbers. Each problem had two possible answers: One correct and one incorrect. The incorrect possible answer was within the range of the correct answer plus or minus 3. The problem and two possible answers were simultaneously presented on the screen, with the problem at the top and the answers at the bottom. Subjects were asked to press the “Q” key if the left-side answer was correct or the “P” key otherwise. The problem and possible answers did not disappear until the participant responded. Participants were encouraged to respond as quickly as possible without sacrificing accuracy. The test was limited to 2 min.

#### Numerosity Comparison

This test investigated the participant’s ability to navigate the ANS. For each trial, two dot arrays were presented simultaneously on the screen and the participant was required to judge which dot array contained more dots while ignoring all other visual properties of the arrays. If participants judged that the left dot array contained more dots, they were cued to press “Q” on the keyboard (or to press “P” otherwise). The number of dots in each array ranged from 5 to 32 to exclude numbers in the subitizing range (1–4 dots).

The dots in an array were randomly distributed within a circle and their sizes varied. The ratios between numbers of dots in each array ranged from 1:12 to 2:0. Gebuis and Reynvoet (2011) proposed that in numerosity comparison, five visual properties need to be controlled: Total surface area, envelope area or convex hull, item size, density (envelope area divided by total surface), and circumference. A previous study showed that performance on numerosity comparison tasks remains ratio-dependent after ruling out the variance of the five key visual properties by partial correlation analysis (Zhou et al., 2015).

There were 120 trials in the test. The trials were divided into six difficulty levels according to the ratio of number of dots in the two arrays (more:fewer) from largest to smallest. The participants started the test at the simplest level. If their percentage of correct answers was larger than or equal to 75%, they advanced to the more difficult level; otherwise they continued at an easier level (or stayed at the same level if they were already at the easiest level). Participants were required to finish 40 trials. A recent study suggested that common measures of the Weber fraction (w) are reliable only when using a substantial number of trials; the researchers found that more than 600 trials were needed to reach an acceptable reliability of 0.8, even under ideal conditions (Lindskog et al., 2013). The purported indirect measure of ANS acuity in terms of the Numeric Distance Effect (NDE) was not reliable and showed no sign of predictive validity (Lindskog et al., 2013), so our final scores were calculated as the sum of weighted scores in each level, which was the number of correctly answered questions times the difficulty level (1–6).

#### Figure Matching

This task assessed the participant’s visual form perception capability. It was adapted from Ekstrom et al. (1976) identical picture test in the Manual of Factor-Referenced Cognitive Tests. For each trial, one image was presented on the left side of the screen while three images were presented on the right. Each picture was a combination of two simple line figures randomly selected from 150 simple line figures. The pictures were first presented for a varied interval from 300 ms to 1600 ms, followed by a 1000-ms blank screen. The participants were required to press “Q” if they judged that any of the pictures on the right side matched the picture on the left and “P” if they did not.

The test difficulty was defined according to the presentation time. The longer the stimulus was presented, the easier the trial was. There were 14 levels, varying from 300 ms to 1600 ms, with a 100-ms difference between levels. The test started at the simplest level (1600 ms). If participants’ correct percentage was larger than or equal to 75%, they advanced to the more difficult level; otherwise, they returned to the easier level (or stayed at the same level if they were already at the easiest level).

Participants were required to finish 40 trials. The final score was calculated as the sum of weighted scores in each level, which was the number of correctly answered questions times the difficulty level between 1 and 14, where the 1600-ms presentation corresponded to a score of 1 and the 300-ms presentation corresponded to a score of 14.

#### Mental Rotation

This test assessed spatial processing ability and was adapted from Shepard and Metzler’s (1971) mental rotation task. In each trial, a three-dimensional figure was presented on the top of the screen and another two three-dimensional figures were presented beneath it. Participants were asked to judge which figure from the bottom pair was the same as the top figure after it was rotated. The rotation angles varied from 15° to 345°. Participants pressed “Q” if their choice was the bottom left figure and “P” otherwise. This test included 180 trials and lasted 3 min.

#### Non-verbal Matrix Reasoning

The test assessed general intelligence and was designed similar to Raven’s Progressive Matrices (Raven, 2000). Participants were asked to complete a picture presented on a computer screen by clicking on the missing corner portion of the image from five candidate portions. The test had 36 trials and stopped when five total trials were incorrect.

#### Choice Reaction Time

This test assessed processing speed and its split-half reliability was found to be 0.84 (Wei et al., 2012b). For all 30 trials, a fixation “+” was first presented on the middle of the screen with a white dot either on the left or right of the fixation cross. Participants pressed “Q” if the white dot appeared on the left side or “P” if it appeared on the right. The inter-stimulus interval was randomly determined between 1500 and 3000 ms. Each participant’s reaction time and accuracy were recorded.

The measure indexes for all eight tests are displayed in **Table 1**. For the tests of subtraction and mental rotation, the adjusted numbers of correct trials were used as scores. The adjusted number of correct trials was calculated as the difference between the numbers of correct and incorrect responses, which was used to control for guessing (Salthouse, 1994; Salthouse and Meinz, 1995; Hedden and Yoon, 2006; Cirino, 2011). This procedure followed the Guilford correction formula S = R-W/(n-1), where S is the adjusted number of items that participants can perform without the aid of chance, R is the number of correct responses, W is the number of incorrect responses, and n is the number of alternative responses for each item (Guilford and Guilford, 1936). This procedure has been used in several recent studies on mathematical cognition (Cirino, 2011; Wei et al., 2012a,b) and general cognition (Salthouse, 1994; Putz et al., 2004; Hedden and Yoon, 2006).

**Table 1:** Means and standard deviations of test scores on index of 8 tests and tests’ reliability coefficients.

Test	Index	Mean (SD)	Split-half reliability
(1) Graded mathematical achievement	Score	11.4 (5.8)	0.83
(2) Semester-final examination	Score (0–100)	88.8 (7.6)	–
(3) Subtraction	Adj. No. of correct trials	34.2 (10.8)	0.90
(4) Numerosity comparison	Score	90.4 (50.7)	0.99
(5) Figure matching	Score	214.2 (149.2)	0.99
(6) Mental rotation	Adjust no. of correct trials	12.3 (11.2)	0.91
(7) Non-verbal matrix reasoning	Adjust no. of correct trials	4.9 (2.9)	0.86
(8) Choice reaction time (ACC)	Accuracy (%)	91.9 (12.8)	0.86
Choice reaction time (RT)	Reaction time (millisecond)	438.6 (171.9)	0.93

*Adj.: adjusted. No.: number. Adj. No. of correct trials = total correct trials minus total incorrect trials. This adjustment was made to control for the effect of guessing in multiple choice tests. ACC: accuracy rate; RT: reaction time.*

For the graded, numerosity comparison, and figure matching tests, the mean score on all responded trials was used as the final score. The trials for the three tests were weighted according to their difficulty or grade. The scores were equal to the sum of the number of correct trials across the different difficulty levels multiplied by the corresponding difficulty level. The non-verbal matrix reasoning task was stopped after five incorrect trials, and the number of correct trials was used as the score. For the choice reaction time task, the median reaction time and accuracy were used as the final scores.

### Procedure

The full battery of tests (except for the curriculum-based, semester end exam) was administered in 60 min. Tests were administered to participants in each class (20–30 participants per class) under the experimentor’s supervision. The testing was conducted in a quiet computer room. For each test, a practice session (four to six trials) accompanied by instruction was conducted just prior to the formal testing session. The tests were administered in the same order for all participants. For the self-adapted math achievement test, participants were asked to click a mouse to choose the correct answer. For all other tests, participants indicated their responses by pressing one of two keys (“P” or “Q”) on the computer keyboard with the index finger of each hand.

Students’ responses and reaction times were automatically recorded and transmitted over the Internet to a server located in a laboratory at Beijing Normal University. All data were collected between April 5 and June 1, 2015.

### Data Analyses

Inter-correlation analyses were first conducted on all measures, then a series of hierarchy regression analyses were performed to test the influence of ANS and figure-matching acumen on math achievement and subtraction capability after controlling for age, gender, and other general cognitive processes.

## Results

The means and standard deviations of all test scores are shown in **Table 1**. All the tests had acceptable split-half reliabilities (0.83–0.99), which were computed from the data of the current study.

### Inter-Correlations between All Measures

Pearson’s correlation coefficients between all measures are displayed in **Table 2**. The graded mathematical achievement score was significantly correlated with subtraction, numerosity comparison, figure matching, mental rotation, and non-verbal matrix reasoning scores. The semester-final examination score was significantly correlated with subtraction and numerosity comparison scores.

**Table 2:** Intercorrelations among all measures.

Test	1	2	3	4	5	6	7	8
(1) Graded mathematical achievement	–							
(2) Curriculum-based math	0.14*	–						
(3) Subtraction	0.44***	0.30***	–					
(4) Numerosity comparison	0.23***	0.13*	0.22***	–				
(5) Figure matching	0.35***	0.10	0.35***	0.42***	–			
(6) Mental rotation	0.20***	0.03	0.27***	0.20**	0.20**	–		
(7) Non-verbal matrix reasoning	0.38***	0.13	0.41***	0.20**	0.35***	0.27***	–	
(8) Choice reaction time (ACC)	0.05	0.05	0.13	0.01	0.20**	0.03	0.14*	–
Choice reaction time (RT)	-0.02	-0.04	-0.21**	0.03	-0.14*	-0.02	-0.10	-0.40***

*^∗^*p* < 0.05, ^∗∗^*p* < 0.01, ^∗∗∗^*p* < 0.001. ACC: accuracy rate; RT: reaction time.*

### Hierarchical Regression Analyses

We conducted hierarchical regression analyses further investigate the role of ANS and figure matching acuity in mathematical achievement and fluency. The results are displayed in **Tables 3** and **4**. According to **Table 3**, when controlling for the three general cognitive processes (choice reaction time, mental rotation, non-verbal matrix reasoning) as well as gender and age, numerosity scores still accounted for 1.9% of the variance in graded mathematical achievement (*F* = 5.05, *p* = 0.026). When controlling for the three general cognitive processes as well as gender and age, the relation between numerosity scores, and semester-final examination was no longer significant (*F* = 1.28, *p* = 0.259). When controlling for the three general cognitive processes as well as gender and age, numerosity scores still accounted for 1.5% of the variance in subtraction scores (*F* = 4.12, *p* = 0.044).

**Table 3:** Results from hierarchical multiple regression analysis for the relations of numerosity comparison and math achievement.

Predictors	Step 1	Step 2	Step 3
	
	β	β	β
**Graded mathematical achievement**			
Age	-0.05	-0.06	-0.07
Gender	0.06	0.08	0.05
Choice reaction time (ACC)	-	-0.02	-0.04
Choice reaction time (RT)	-	0.01	-0.00
Mental rotation	-	0.12	0.09
Non-verbal matrix reasoning	-	0.36*^∗∗∗^*	0.34*^∗∗∗^*
Numerosity comparison	-	-	0.15*^∗^*
	*R^2^= 0.01*	*ΔR^2^= 0.16^∗∗∗^*	*ΔR^2^= 0.02^∗^*
**Semester-final examination**			
Age	-0.01	-0.01	-0.02
Gender	0.23*^∗∗^*	0.24*^∗∗∗^*	0.22*^∗∗^*
Choice reaction time (ACC)	-	-0.02	-0.03
Choice reaction time(RT)	-	-0.06	-0.07
Mental rotation	-	0.03	0.02
Non-verbal matrix reasoning	-	0.12	0.11
Numerosity comparison	-	-	0.08
	*R^2^= 0.05^∗∗^*	*ΔR^2^= 0.02^∗^*	*ΔR^2^= 0.01*
**Subtraction**			
Age	0.01	0.01	-0.00
Gender	-0.02	0.02	-0.00
Choice reaction time (ACC)	-	0.00	-0.04
Choice reaction time (RT)	-	-0.18*^∗∗^*	-0.18*^∗∗^*
Mental rotation	-	0.18*^∗∗^*	0.15*^∗^*
Non-verbal matrix reasoning	-	0.34*^∗∗∗^*	0.32*^∗∗∗^*
Numerosity comparison	-	-	0.13*^∗^*
	*R^2^= 0.001*	*ΔR^2^= 0.22^∗∗∗^*	*ΔR^2^= 0.02^∗^*

*^∗^*p* < 0.05, ^∗∗^*p* < 0.01, ^∗∗∗^*p* < 0.001. ACC: accuracy rate; RT: reaction time.*

**Table 4:** Results from hierarchical multiple regression analysis for the relations of figure matching and math achievement.

Predictors	Step 1	Step 2	Step 3	Step 4
	
	β	β	β	β
**Graded mathematical achievement**				
Age	-0.05	-0.06	-0.04	-0.05
Gender	0.06	0.08	0.09	0.08
Choice reaction time (ACC)	–	-0.02	-0.05	-0.06
Choice reaction time (RT)	–	0.01	0.04	0.01
Mental rotation	–	0.12	0.09	0.08
Non-verbal matrix reasoning	–	0.36*^∗∗∗^*	0.28*^∗∗∗^*	0.28*^∗∗∗^*
Figure matching	–	–	0.25*^∗∗∗^*	0.22*^∗∗^*
Numerosity comparison			–	0.06
	*R^2^= 0.01*	*ΔR^2^= 0.16^∗∗∗^*	*ΔR^2^= 0.05^∗∗∗^*	*ΔR^2^= 0.00*
**Semester-final examination**				
Age	-0.01	-.01	-.00	-0.01
Gender	0.23*^∗∗^*	0.24*^∗∗∗^*	0.24*^∗∗∗^*	0.23*^∗∗^*
Choice reaction time (ACC)	–	-0.02	-0.03	-0.03
Choice reaction time (RT)	–	-0.06	-0.06	-0.06
Mental rotation	–	0.03	0.02	0.01
Non-verbal matrix reasoning	–	0.12	0.10	0.09
Figure matching	–	-	0.08	0.05
Numerosity comparison			–	0.06
	*R^2^= 0.05^∗∗^*	*ΔR^2^= 0.02^∗^*	*ΔR^2^= 0.01*	*ΔR^2^= 0.00*
**Subtraction**				
Age	0.01	0.01	0.02	0.02
Gender	-0.02	0.02	0.04	0.03
Choice reaction time (ACC)	–	0.00	-0.03	-0.03
Choice reaction time (RT)	–	-0.18*^∗∗^*	-0.17*^∗^*	-0.16*^∗∗^*
Mental rotation	–	0.18*^∗∗^*	0.15*^∗^*	0.15*^∗^*
Non-verbal matrix reasoning	–	0.34*^∗∗∗^*	0.28*^∗∗∗^*	0.27*^∗∗∗^*
Figure matching	–	–	0.21*^∗∗^*	0.19*^∗∗^*
Numerosity comparison			–	0.06
	*R^2^= 0.00*	*ΔR^2^= 0.22^∗∗∗^*	*ΔR^2^= 0.04^∗∗^*	*ΔR^2^= 0.00*

*^∗^*p* < 0.05, ^∗∗^*p* < 0.01, ^∗∗∗^*p* < 0.001. ACC: accuracy rate; RT: reaction time.*

According to **Table 4**, when controlling for the three general cognitive processes as well as gender and age, visual form perception still accounted for 5.2% of the variance in graded mathematical achievement scores (*F* = 13.98, *p* < 0.001). When controlling for the three general cognitive processes as well as gender and age, the relation between visual form perception and semester-final examination was no longer significant (*F* = 1.13, *p* = 0.289). When controlling for the three general cognitive processes as well as gender and age, visual form perception still accounted for 3.8% of the variance in math fluency (*F* = 10.77, *p* = 0.001). For the three dependent measures, the numerosity comparison scores, as the fourth step, did not explain any further variance.

## Discussion

In this study, we found that the relation between ANS acuity and mathematical achievement is indeed influenced by fluency. Three types of mathematical achievement tests were used to gather relevant data: Subtraction, graded test (first to 12th grades) and semester-final examination. We first replicated the previous finding on the relation between ANS acuity and math fluency (Halberda et al., 2008; Zhou and Cheng, 2015; Zhou et al., 2015; Zhang et al., 2016). We found that graded mathematical achievement, but not semester-final examination score, is still significantly correlated with numerosity comparison score when controlling for age, gender, and general cognitive processes (choice reaction time, mental rotation, non-verbal matrix reasoning). Furthermore, the variance in graded mathematical achievement contributed by numerosity comparison can be interpreted via the visual form perception scores. These results conform to our expectations prior to conducting this study.

Previous studies have shown a dissociation between subtraction and mathematical problem-solving (Geary et al., 2000; Inglis et al., 2011; Lyons and Beilock, 2011; Wei et al., 2012b; Zhou and Cheng, 2015; Zhou et al., 2015). In this study, we found that the mathematical fluency may be the cause of said dissociation; that is, ANS acuity is only correlated with the mathematical performance involving mathematical fluency as opposed to solving problems. The participant’s performance on math fluency tests is likely dependent on his or her automatic retrieval of math facts and procedures, (assuming that he or she is proficient in them), such as addition, subtraction, and multiplication (Zhou and Cheng, 2015; Zhou et al., 2015). Conversely, the participant is dependent on step-by-step strategies to solve math problems such as number series completion tasks (Woodcock et al., 2001; Inglis et al., 2011; Wei et al., 2012b; Zhang et al., 2016).

Previous studies have showed inconsistence in the relation between ANS acuity and mathematical achievement using the same mathematical tests for participants of various ages. For example, some studies failed to find significant correlations between ANS acuity and arithmetic performance for children using the Woodcock-Johnson III Test of Achievement (Inglis et al., 2011; Price et al., 2012). Other studies also using the Woodcock-Johnson III Test of Achievement showed that calculation score is significantly correlated with ANS acuity in adults (Lourenco et al., 2012; Agrillo et al., 2013). This disparity can be attributed to the fact that adult participants express a greater extent of mathematical fluency than child participants in the same subtraction test; adults have more practice with retrieving the information necessary for subtraction (i.e., have honed their mathematical fluency), while children tend to use slower reasoning strategies with their relatively newly acquired math skills to solve the same problems.

Studies have shown that the relation between ANS acuity and subtraction can be accounted for by domain-general visual form perception performance (Zhou and Cheng, 2015; Zhou et al., 2015). The results presented here support the assertion that ANS acuity and mathematical achievement cover a wide range of math knowledge, rather than being limited to subtraction, which can also be accounted for by general visual form perception performance. The visual form processing of mathematical symbols may play a substantial role in mathematical achievement test performance.

This study was not without limitations. First, a relatively small range of participants were assessed. The participants were fifth graders from local primary schools, so our results may not be generalizable to children in other age groups. Future research should include participants from different grades in primary schools and middle schools. Second, although the questions on the graded mathematical achievement test were from the semester-final examination, they differed in several dimensions such as the number of questions and the range of mathematical knowledge involved. Future studies should directly manipulate mathematical fluency while controlling for the related factors in the mathematical achievement tests.

Taken together, our findings suggest that the relation between ANS acuity and mathematical achievement is influenced by fluency. In other words, ANS acuity and visual form perception are correlated with mathematical achievement in regards to math fluency. The underlying mechanism for the relation between ANS acuity and mathematical achievement may be characterized by general visual form perception.

## Author Contributions

XZ developed the study concept and completed the study design. LW and YS collected data. LW performed the data analysis and interpretation under the supervision of XZ. LW drafted the manuscript, XZ and YS provided critical revisions. All authors approved the final version of the manuscript for submission.

## Conflict of Interest Statement

The authors declare that the research was conducted in the absence of any commercial or financial relationships that could be construed as a potential conflict of interest.

## Footnotes

1 www.dweipsy.com/lattice
